# A human expression system based on HEK293 for the stable production of recombinant erythropoietin

**DOI:** 10.1038/s41598-019-53391-z

**Published:** 2019-11-14

**Authors:** Christine Lin Chin, Justin Bryan Goh, Harini Srinivasan, Kaiwen Ivy Liu, Ali Gowher, Raghuvaran Shanmugam, Hsueh Lee Lim, Matthew Choo, Wen Qin Tang, Andy Hee-Meng Tan, Terry Nguyen-Khuong, Meng How Tan, Say Kong Ng

**Affiliations:** 1 Bioprocessing Technology Institute, Agency for Science, Technology and Research (A*STAR), Singapore, Singapore; 2 School of Chemical and Biomedical Engineering, Nanyang Technological University, Singapore, Singapore; 3 Genome Institute of Singapore, Agency for Science, Technology and Research (A*STAR), Singapore, Singapore

**Keywords:** Genetic engineering, Expression systems, Transcriptomics

## Abstract

Mammalian host cell lines are the preferred expression systems for the manufacture of complex therapeutics and recombinant proteins. However, the most utilized mammalian host systems, namely Chinese hamster ovary (CHO), Sp2/0 and NS0 mouse myeloma cells, can produce glycoproteins with non-human glycans that may potentially illicit immunogenic responses. Hence, we developed a fully human expression system based on HEK293 cells for the stable and high titer production of recombinant proteins by first knocking out *GLUL* (encoding glutamine synthetase) using CRISPR-Cas9 system. Expression vectors using human *GLUL* as selection marker were then generated, with recombinant human erythropoietin (EPO) as our model protein. Selection was performed using methionine sulfoximine (MSX) to select for high EPO expression cells. EPO production of up to 92700 U/mL of EPO as analyzed by ELISA or 696 mg/L by densitometry was demonstrated in a 2 L stirred-tank fed batch bioreactor. Mass spectrometry analysis revealed that N-glycosylation of the produced EPO was similar to endogenous human proteins and non-human glycan epitopes were not detected. Collectively, our results highlight the use of a human cellular expression system for the high titer and xenogeneic-free production of EPO and possibly other complex recombinant proteins.

## Introduction

Mammalian cells have been used for the production of recombinant protein therapeutics for over three decades to-date due to the requirement of post-translational modifications (PTMs) for activity^1^. Thus far, mammalian cell lines approved for the manufacture of recombinant protein therapeutics include Chinese hamster ovary (CHO) cells, baby hamster kidney (BHK) cells, Sp2/0 murine hybridoma cells, NS0 murine myeloma cells, C127 murine mammary gland cells, HT-1080 human fibrosarcoma cells and human embryonic kidney cells 293 (HEK293)^2^. CHO cells are the dominant mammalian host cell lines used for the manufacture of recombinant protein therapeutics^1^ due to a number of advantages: (1) CHO cells confer human-like glycosylation features to glycoprotein products, (2) are capable of high protein productivity with titers up to grams per liter due to established stable cell line generation strategies, (3) propagate well in serum-free single-cell suspension cultures enabling ease of scale-up, (4) are refractory to a number of human viruses providing lower biosafety risks, and (5) have established regulatory track records for the manufacture of recombinant protein therapeutics^3–6^. Nevertheless, CHO cells have been reported to generate non-human glycan structures including N-glycolylneuraminic acid (Neu5Gc) and galactose-alpha-1,3-galactose group (α-Gal) onto glycoprotein products which confers increased risk of immunogenicity^7,8^. CHO-derived glycoproteins also lack certain human glycans, including bisecting GlcNAc, Lewis^x^, sialyl Lewis^x^, and α(2,6)-linked sialic acid^6,8^. Moreover, CHO cells may not adequately produce other PTMs (such as glutamic acid γ-carboxylation) required for activity of some recombinant protein therapeutics^9^.

In efforts to obtain more human-like protein therapeutics, pharmaceutical companies have utilized various human cell lines, such as HEK293, HT-1080, AGE1.HN, CAP, HKB-11 and PER.C6 for glycoprotein production^2,8^. While products from AGE1.HN, CAP, HKB-11 and PER.C6 are still at different phases of preclinical and clinical development, several protein therapeutics from HEK293 and HT-1080, including recombinant factor VIII-Fc, Dulaglutide, Idursulfase and Velaglucerase alfa have already been approved by the US Food and Drug Administration (FDA) and European Medicines Agency (EMA)^2^. These regulatory approvals for HEK293 and HT-1080 demonstrate successful circumvention of potential biosafety risks from human virus contamination and provide a useful basis for future assessment of other therapeutics produced using human cell lines.

HEK293 cells are a particularly attractive expression system for recombinant protein production as they offer multiple advantages besides being able to generate human glycosylation profiles and having an established regulatory track record. First, it is exceptionally efficient in glutamic acid γ-carboxylation and tyrosine sulfation, which is required of some therapeutic products like Drotrecogin alfa and recombinant factor IX-Fc^2,9–11^. Second, it is easily manipulated and can be used for rapid production of recombinant proteins via transient gene expression^12,13^. Third, it can be used for stable recombinant protein production, which has been reported for IFNα2b using HEK293-6E cells with titers of up to 333 mg/L^14^ and antibodies using HEK293F cells with titers of up to 600 mg/L^15^.

Despite the potential of HEK293 as a human bioproduction cell line, stable recombinant protein production in HEK293 has been achieved mainly using xenogeneic genes as selection markers. For example, HEK293-6E cells constitutively expressing the non-native Epstein–Barr virus (EBV) EBNA1 protein have been utilized for the amplification of expression vectors with a suitable viral origin of replication^14^. Antibiotic selection strategies involving resistance cassettes from other organisms have also been deployed. The usage of xenogeneic genes will result in higher expression of non-human proteins in the HEK293 producer cell lines on top of the E1 adenoviral genes that the HEK293 expresses, and potentially trace amounts of these proteins can contaminate the final product. Such contamination may trigger allergic and adverse reactions, and/or immunogenicity to the product.

To offer a bioproduction system with low expression of heterologous proteins, we explored the use of human glutamine synthetase (*GLUL*) gene as a selection marker for the development of producer cell lines. Glutamine is an amino acid required for the biosynthesis of several amino acids, pyrimidines and purines, and it can be synthesized from glutamate and ammonia by GLUL or can be exogenously supplemented to culture medium to support cell growth. As a selection marker, GLUL expression becomes essential in the absence of glutamine in the culture media. Coupled with the expression of the transgene-of-interest and upon addition of sub-lethal doses of methionine sulfoximine (MSX), a GLUL inhibitor, cells expressing high levels of GLUL with simultaneously increased expression of the target transgene were selected for. This GLUL-mediated gene selection strategy was first demonstrated in CHO cells^16^, and subsequently implemented in HEK293E cells but with low titers^17,18^. In this study, we knocked out the endogenous *GLUL* gene in HEK293 cells using the CRISPR-Cas9 system, characterized the cells by RNA sequencing (RNA-seq), and demonstrated the utility of our bioproduction platform for the production of human erythropoietin (EPO) as a model product. High producer cells, selected using MSX in glutamine-deficient media, were characterized in batch shake flask and fed-batch bioreactor cultures.

## Results

### Inactivation of *GLUL* in HEK293 cells using CRISPR-Cas9

**Figure 1:**
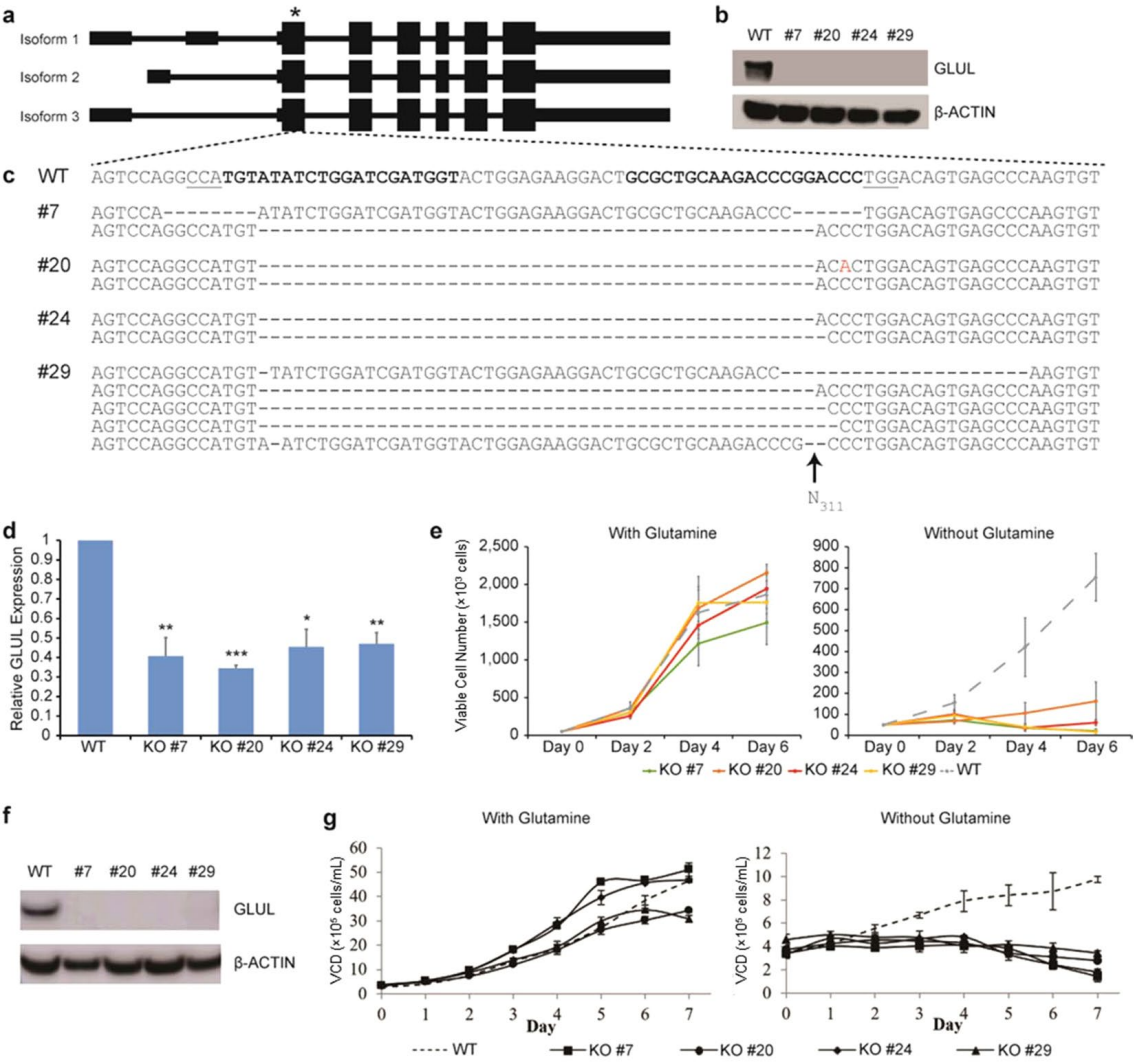
Generation of HEK293 *GLUL* knockout (KO) cells. (**a**) Schematic of the three *GLUL* isoforms. HEK293 wildtype (WT) cells were transfected with vectors encoding Cas9 and two gRNAs targeting the first constitutive protein-coding exon of the *GLUL* gene. The target site is indicated with an asterisk. (**b**) Immunoblots showing the presence of GLUL protein in wildtype cells, but absence of protein in four isolated KO clones, cultivated as adherent cultures. (**c**) *GLUL* sequence at the target site. The spacer sequences of the gRNAs are indicated in bold, while the protospacer adjacent motifs (PAMs) of Cas9 from *Streptococcus pyogenes* (SpCas9) are underlined. The two gRNAs target opposite strands of the genomic DNA. (**d**) Relative expression of GLUL in WT and KO cells, as assayed by qPCR. Values represent mean ± S.E.M. (*P < 0.05, **P < 0.01 ***P < 0.001; Student’s t-test) (**e**) Sensitivity of WT and KO cells to glutamine-deficient media. WT cells are indicated by a dotted line, while the four KO clones are indicated by solid colored lines. The cells were grown in adherent culture conditions. Values represent mean ± S.E.M. (**f**) Immunoblots showing the presence of GLUL protein in wildtype cells, but absence of protein in four isolated KO clones cultivated in suspension culture conditions. (**g**) Sensitivity of WT and KO cells to glutamine-deficient media. WT is represented in a broken line, while *GLUL*-KO #7 (square), #20 (circle), #24 (diamond), and #29 (triangle) are depicted in solid lines and symbols. The cells were grown in suspension culture conditions. Values represent mean ± SD.

In order to prevent endogenous GLUL protein from interfering with our gene selection strategy as observed in a previous report^17^, we sought to knock out the native *GLUL* gene in HEK293 using the CRISPR-Cas9 system. Two guide RNAs (gRNAs) were designed to target the first constitutive protein-coding exon (Fig. 1a) which would inactivate all isoforms simultaneously. Following transfection with the Cas9 and gRNA plasmids, we selected for the successfully transduced cells by flow cytometry and then plated the sorted cells sparsely on a plate to allow single cells to grow up as individual colonies. After picking and expanding multiple individual clones, we screened all of them for loss of GLUL protein by Western blot and identified four clones where the protein was absent (Fig. 1b). Subsequently, we sequenced the target genomic locus of the four clones. For clones #7, #20, and #24, two distinct alleles were found in each of them (Fig. 1c). In clone #7, we detected one allele with 14 bp deletion and another allele with 47 bp deletion; in clone #20, we uncovered two different 47 bp deletions; and in clone #24, we detected one allele with 47 bp deletion and another allele with 48 bp deletion. Lastly, for clone #29, we uncovered five distinct alleles (Fig. 1c), suggesting that the clone may have grown a merged colony containing two or more single cells. All observed mutations except the 48 bp deletion resulted in frameshifts, which may trigger nonsense-mediated decay of the GLUL transcript^19^. Consequently, gene expression analysis by quantitative real-time PCR (qPCR) showed that GLUL transcript levels were indeed significantly down-regulated in all four clones (Fig. 1d). To verify the loss of GLUL function in our knockout clones, we monitored the growth rates of the cells in media either supplemented with or deficient of glutamine. Glutamine dependency screening was previously used in CHO, NS0 and HEK293E cell lines to identify clones lacking active GLUL protein^18,20^. Here, we observed that there was no clear difference in growth rate between wildtype HEK293 cells and all the *GLUL*^−/−^ clones in glutamine-supplemented media with all the cell lines reaching over 90% confluency within 6 days (Fig. 1e, left panel). However, our generated *GLUL*^−/−^ clones were unable to proliferate in glutamine-deficient media, unlike the original wildtype HEK293 cells (Fig. 1e, right panel), thereby verifying that we had successfully inactivated the *GLUL* gene.

As part of the process to utilize our novel *GLUL*^−/−^ cell lines for bioproduction, we adapted them to suspension serum-free culture together with the wildtype HEK293 cells. Immunoblot analysis at this stage again confirmed that the GLUL protein was absent in our knockout clones (Fig. 1f). We also characterized the growth profiles of the cells in culture media with or without glutamine (Fig. 1g). Overall, wildtype and *GLUL*^−/−^ cells propagated slower in suspension compared to adherent culture. In glutamine-supplemented media, *GLUL*^−/−^ cells clones #7 and #24 grew slightly faster than wildtype HEK293 cells, while clones #20 and #29 had similar growth profiles compared to wildtype HEK293 cells, with the various cell lines reaching viable cell densities (VCD) of approximately 3 to 5 × 10^6^ cells/mL on day 6 of the batch culture (Fig. 1g, left panel). In glutamine-deficient media, while we observed slower growth for wildtype HEK293 cells (reaching cell densities of only 1 × 10^6^ cells/mL at day 7), all four *GLUL*^−/−^ clones were unable to proliferate (Fig. 1g, right panel), thereby confirming the functional loss of *GLUL* in these knockout cell lines.

### Transcriptome analysis of *GLUL* knockout cell lines

**Figure 2:**
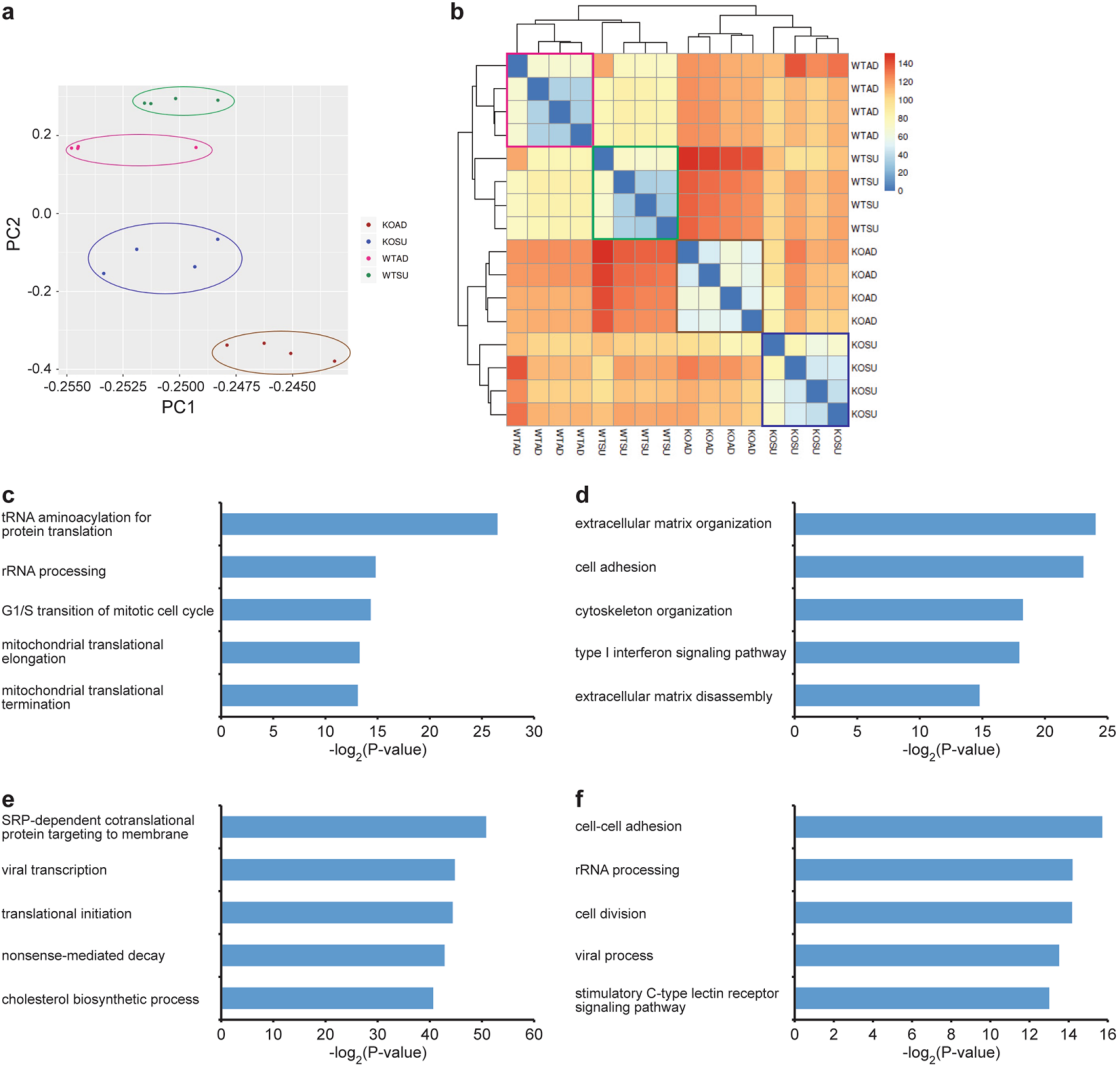
Transcriptome analysis of HEK293 WT and *GLUL*-KO cells. (**a**) PCA of gene expression levels. Four biological replicates were generated for the original WT cells grown under each culture condition (adherent or suspension). (**b**) Hierarchical clustering of our gene expression data. The heatmap showed that the various samples first separated based on *GLUL* gene status (i.e. *GLUL*^+/+^ or *GLUL*^−/−^) and then by culture condition (adherent or suspension). The values of the heatmap are Euclidean distances. (**c**,**d**) GO analysis of the genes that were significantly (**c**) up-regulated or (**d**) down-regulated in our KO clones when compared to the unmodified WT cells (adjusted P < 0.0001). (**e**,**f**) GO analysis of the genes that were significantly (**e**) up-regulated or (**f**) down-regulated in suspension cells when compared to adherent cells (adjusted P < 0.0001).

To gain insights into the molecular changes in our *GLUL* knockout clones during adherent and suspension culture, we analyzed their transcriptomes by RNA-seq on the Illumina platform. We sequenced poly(A)-selected RNAs isolated from all four *GLUL*^−/−^ cell lines and the wildtype HEK293 cells from adherent and suspension cultures during exponential phase, and obtained between 19 to 147 million paired-end reads for each sample. Expression counts were calculated using DESeq2^21^. Principal component analysis (PCA) (Fig. 2a) and clustering analysis based on Euclidean distance (Fig. 2b) revealed that the samples separated by experimental conditions and that the differences between wildtype and knockout cells were larger than the differences between adherent and suspension cultures.

First, we compared all the wildtype samples against all the *GLUL*^−/−^ samples, pairing the culture conditions in our analysis, where 915 and 1,142 genes were significantly up-regulated and down-regulated in the *GLUL*^−/−^ cells respectively (adjusted P-value < 0.0001) (Supplementary File 1). Interestingly, Gene Ontology (GO) analysis using DAVID^22^ revealed that the up-regulated genes were significantly enriched for functions related to protein translation (Fig. 2c), possibly in response to the loss of *GLUL*, a key enzyme involved in amino acid metabolism. Additionally, the down-regulated genes were significantly enriched for functions related to the extracellular matrix, cell adhesion, cytoskeleton, and interferon signaling (Fig. 2d).

Second, we compared all the adherent samples against all the suspension samples, pairing the *GLUL* gene status in our analysis- 549 and 596 genes were significantly up-regulated and down-regulated in the cells grown in suspension culture respectively (adjusted P-value < 0.0001) (Supplementary File 2). GO analysis using DAVID^22^ revealed that the up-regulated genes were significantly enriched for functions related to the cell membrane and RNA metabolism (Fig. 2e), perhaps reflecting the fact that the cells were no longer attached to a surface and therefore required remodeling of the cell envelope. Expectedly, we also found that the down-regulated genes were enriched for functions related to cell-cell adhesion and cell division (Fig. 2f), which may account for the slower growth rates in suspension cultures. Collectively, our transcriptome analysis uncovered molecular changes that were consistent with observed phenotypes and we also did not detect any strong gene signature that might indicate alterations in protein glycosylation in the *GLUL*^−/−^ cells that might affect their use as a platform for the manufacture of glycoproteins.

### Productivity and production stability of HEK293 EPO-producing cell lines

**Figure 3:**
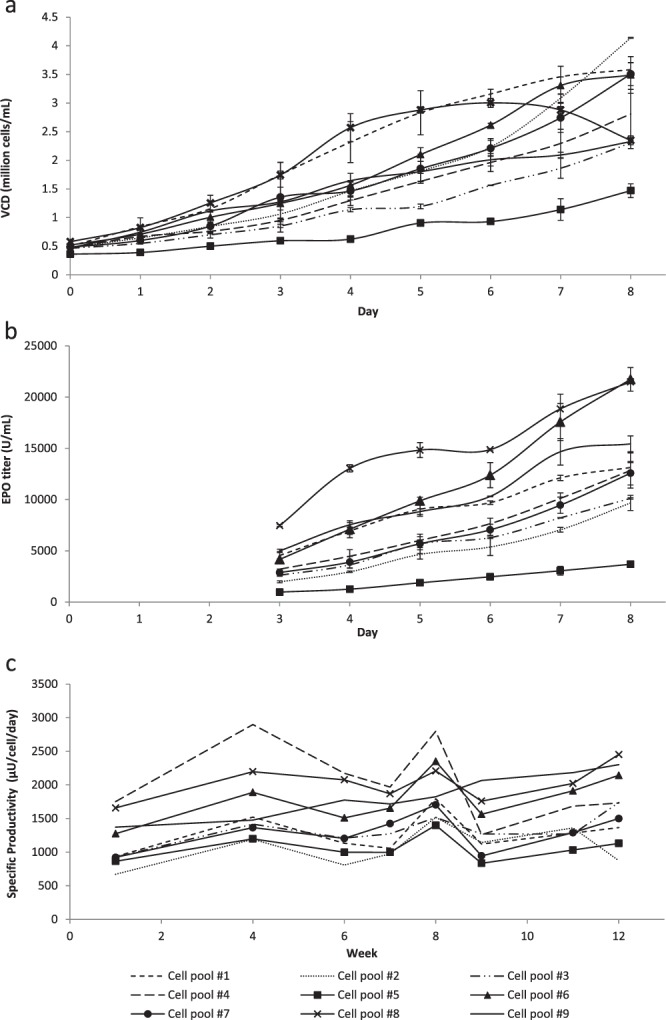
Production and stability of EPO of HEK293 producer cells. *GLUL*-KO clones were transfected with bicistronic vector expressing human GLUL and human EPO cultured in glutamine-deficient media. Post-methionine sulfoximine (MSX) selection, nine cell pools were generated and characterized for (**a**) viable cell density and (**b**) EPO production. Values represent mean ± SD (c) MSX selection was subsequently removed from culture for stability testing over 12 weeks with specific productivity measured at day 4 cultures. (n = 2).

Following the characterization of our *GLUL*^−/−^ cells, we sought to demonstrate their utility in bioproduction, using human EPO as a model product. Knockout cells were transfected with a bicistronic vector expressing human GLUL and human EPO. MSX, a potent inhibitor of GLUL activity, was then used for gene selection and high expression of GLUL-based expression vectors^23,24^. Selection with MSX based on previous reports using CHO and NS0 cell lines span a wide range from 25 µM to 1000 µM^24–27^. For a related study in a HEK293E *GLUL*^−/−^ cell line, 12.5 µM of MSX was used to select for GLUL-based expression vectors to mAb yields of about 8 mg/L with specific productivity of about 4.5 pcd (picogram/cell/day)^18^. In comparison, we were able to obtain high-producer HEK-EPO mini-pools with 100 nM MSX, possibly due to the attenuation of *GLUL* gene in the expression vector^28,29^. A total of 175 mini-pools were generated from the selection process and 9 were subsequently adapted to suspension cultures. In a batch shake flask culture, maximum viable cell densities of these mini pools range from 1.4 × 10^6^ to 4.1 × 10^6^ cells/mL, with EPO titers ranging from 3680 to 21730 U/mL (Fig. 3a,b). The highest producers, cell pools #8 and #6, achieved EPO titers of 21480 and 21730 U/mL and a maximum viable cell density of 3.0 × 10^6^ cells/mL and 3.5 × 10^6^ respectively.

Production stability is an important consideration for selecting a suitable cell line in biotherapeutics manufacturing, especially in view of recent interests in long-term perfusion cultures. Previous studies have conflicting reports on the stability of GLUL-based selection systems, which may be clone dependent: A stability study of over 30 passages carried out in mAb-producing CHO cells showed most clones decreased in productivity when cultured either in the presence or absence of 25–50 µM MSX treatments although stable clones can be obtained^24^. Decrease in productivity was similarly reported in other CHO-mAb producing cell lines, especially when MSX was withdrawn from the culture^27,30,31^. In another report using *GLUL* deficient NS0 cells, recombinant protein production with GLUL-based vectors was demonstrated to be stable in 2 out of 4 clones analyzed over 134 days of passaging^32^.

In this study, the stability of HEK-EPO cell pools were assessed to analyze the general production stability of cells developed using this system. As cell pools are heterogeneous cell populations, these will be representative of large subsets of cells, in contrast to the use of single cell clones which will reflect clonal variability of cell pools they were derived from. The cell pools were passaged in media deficient of MSX over a period of 12 weeks. From VCD and titer analysis of day 4 samples at each selected passage, it was noteworthy to observe that all 9 production cell pools retained production stability throughout the 12-week period (Fig. 3c) with specific productivities ranging from 99% to 187% at week 12 compared to week 1. This suggests that the GLUL-MSX selection system in these HEK293 *GLUL*^−/−^ cells generates production cell lines that are generally stable in recombinant protein productivity. This is interesting in light of challenges to obtain stable clones as described above, because the results suggest that stable clones can be easily obtained using this system. We postulate that this may be due to the low MSX concentration used for selection in this study. Improvements in specific productivities over 12 weeks without MSX were also observed for 8 out of the 9 cell pools. This can be attributed to cell pools adapting well to a less selective culture medium which allowed for better cell growth and increased titer. As cell pool #8 was one of the highest producer throughout the period of 12 weeks with a more stable specific productivity profile, we selected cell pool #8 for further analysis and production of EPO in bioreactor cultures.

### Nucleic acid analysis of EPO-producing cell pool

**Figure 4:**
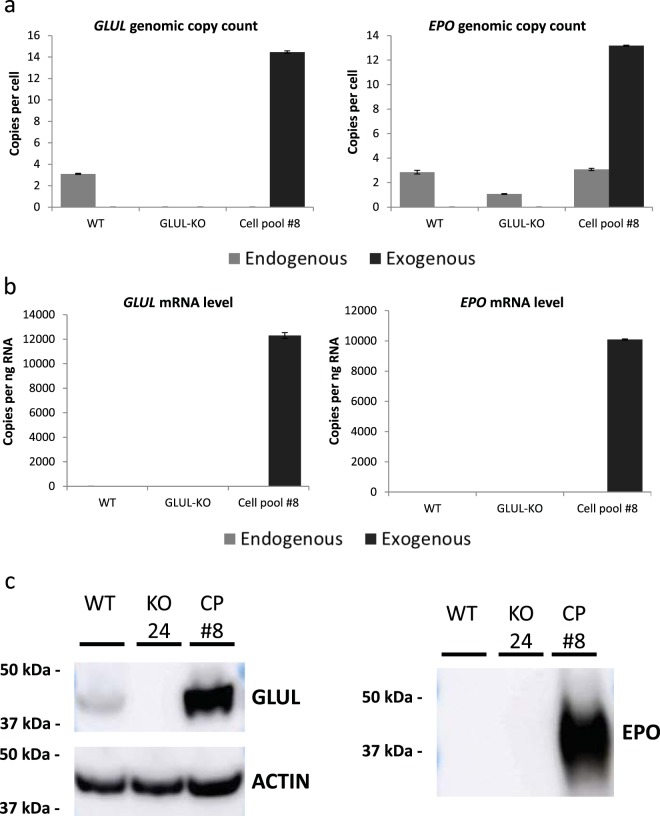
Characterization of EPO expression in GLUL-MSX-mediated HEK293 producer cell pool. (**a**) Endogenous (light gray bars) and exogenous (dark gray bars) *GLUL* and *EPO* genomic DNA copy count analyzed via droplet digital PCR (ddPCR). Values represent mean ± SD (**b**) Endogenous (light gray bars) and exogenous (dark gray bars) GLUL and EPO mRNA copy count analyzed via ddPCR. Values represent mean ± SD (**c**) Immunoblot of GLUL and EPO protein in HEK293 wildtype (WT), *GLUL*-KO (KO #24) and cell pool #8 (CP#8). Actin was used as a loading control.

To characterize cell pool #8, we compare its gene copy number of endogenous and exogenous *GLUL* and *EPO* to that in wildtype and *GLUL*^−/−^ cells via droplet digital PCR (ddPCR). While HEK293 wildtype cells showed basal levels of endogenous *GLUL*, *GLUL*^−/−^ cells showed absence of *GLUL* following disruption with CRISPR-Cas9, since one primer probing for endogenous *GLUL* was designed to be on the gRNA target region. Post GLUL-vector transfection and MSX selection, relative positive ddPCR counts in cell pool #8 showed an exogenous *GLUL* copy number that was four-fold that of the endogenous *GLUL* in wildtype cells (Fig. 4a, left panel). Analysis of *EPO* showed endogenous basal copy numbers in all three cell lines analyzed. For the stably transfected cell pool #8, it showed an exogenous *EPO* level that was about four-fold higher than endogenous *EPO* copy numbers in wildtype cells (Fig. 4a, right panel), fairly consistent with the copy numbers observed for exogenous *GLUL*.

At the transcript level, while we observed low levels of GLUL mRNA expression in wildtype cells at 2.2 copies/ng RNA, practically no expression of GLUL mRNA was detected in *GLUL*^−*/*−^ cells (<0.005 copies/ng RNA) because one primer probing for endogenous *GLUL* was designed to be in the region targeted by the gRNA. Very low expression of EPO mRNA in wildtype and *GLUL*^−/−^ cells (<0.32 copies/ng RNA) was also detected. On the other hand, cell pool #8 showed similar expression levels of exogenous GLUL and EPO mRNA at 12,300 and 10,000 copies per ng RNA respectively, using *RPLP0* as a reference gene (Fig. 4b, left and right panel respectively). These represent a 5,500-fold increase in exogenous GLUL mRNA expression and a 31,000 fold increase in exogenous EPO mRNA expression compared to total expression in wildtype HEK293 cells. The observed increase in GLUL and EPO mRNA expression is significantly greater than the increase in *GLUL* and *EPO* genomic copy number, suggesting the successful selection of a cell pool with transgene insertions into transcriptionally active sites and/or a more robust transcription machinery.

Increased translation to protein was also observed: Immunoblot analysis showed basal expression of the GLUL protein in wildtype HEK293 cells, no expression in the *GLUL*^−/−^ cells and an increased GLUL expression in cell pool #8 (Fig. 4c, left panel). As for EPO protein, expression was only detected in supernatant from cell pool #8 but not in supernatant from wildtype HEK293 and *GLUL*^−/−^ cultures (Fig. 4c, right panel). Taken together, these results from increased gene copy number to final protein expression demonstrated the successful selection of an EPO producing cell pool using our GLUL-MSX selection system comprising of HEK293 *GLUL*^−/−^ cells and expression vector with human *GLUL* selection marker.

### EPO production in fed-batch culture

**Figure 5:**
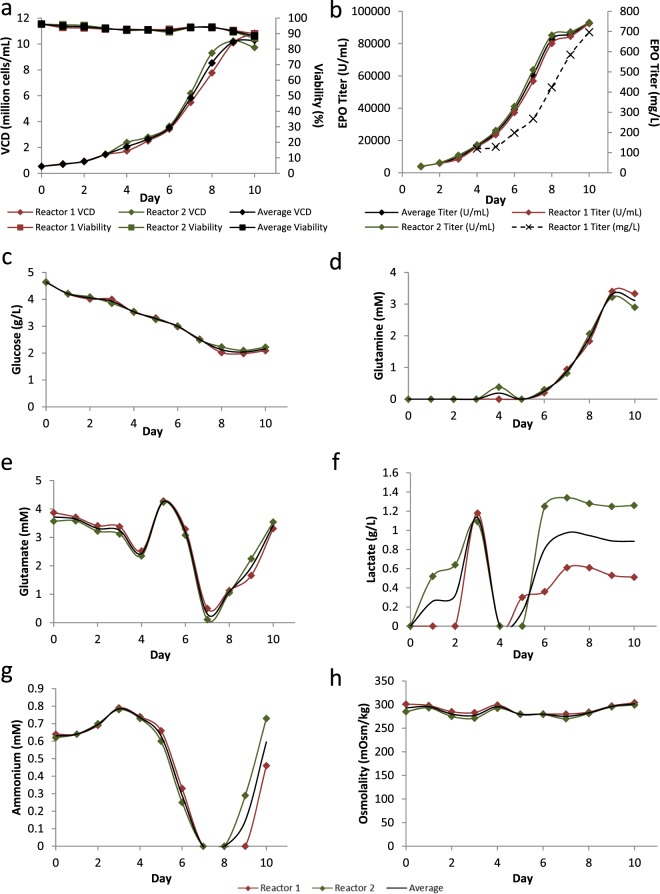
Productivity of HEK293 EPO producer cell pool in a 2 L fed-batch culture. Cell pool #8 was cultured in 2 L stirred-tank bioreactors over 10 days and characterized daily for (**a**) viable cell density (VCD, diamonds) and viability (%, squares). (**b**) EPO production was measured by ELISA over period of culture together with (**c**) glucose (g/L), (**d**) glutamine (mM), (**e**) glutamate (mM), (**f**) lactate (g/L), (**g**) ammonia (mM), and (**h**) osmolality (mOsm/kg).

The ability to achieve effective production of a desired protein in a large-scale manufacture is key for commercial success. To demonstrate potential scalability, we performed a fed-batch culture using HEK-EPO cell pool #8 in a 2 L stirred tank bioreactor (Fig. 5). In the bioreactor, average specific growth rate was 0.0134 h^−1^, reaching average maximum VCD of 10.2 × 10^6^ cells/mL on day 10 (Fig. 5a). Fed-batch culture of the cell pool generated in this study was capable of producing 92700 U/mL of EPO as analyzed by ELISA or 696 mg/L by densitometry (Fig. 5b and Supplementary Fig. 1), with maximum specific productivities of 4070 µU/cell/day or 18.1 pcd. As such, titers achieved in our study are comparatively superior to previous reports on recombinant EPO production (Table 1).

**Table 1:** Comparison of EPO titers.

Reference	Culture characteristics	Max EPO titer
Restelli V *et al*.^50^	Suspension serum free CHO-K1 2.4 L stirred tank culture Culture length: 7 day	2400 U/mL (or 16^*^ mg/L)
Sun *et al*.^51^	Suspension serum free HEK 293 EBNA1 cells cultured in equal volumes of 293 SFM II medium and a 5 × amino acid solution prepared based on DMEM/F12 medium formula Culture length: 10–12 days	4700 U/mL (or 33.6 mg/L)
Wang Z *et al*.^52^	MTX amplified adherent CHO in 12-well plate in IMDM +10% dFBS for 3 days, followed by SFM Culture length: 6 days	8000 U/mL (or 53^*^ mg/L)
Park JH *et al*.^53^	Suspension serum free CHO spinner flask culture Culture length: 6 days	7000 U/mL (or 47^*^ mg/L)
Our results	Suspension serum free HEK293-GS-KO EPO 2 L stirred tank culture supplemented with hydrolysate Culture length: 10 days	92 700 U/mL (or 696 mg/L)

*Estimated conversion based on 150 U/µg, as per the 3rd International Standard by the National Institute for Biological Standards and Control (NIBSC code: 11/170).

Daily analysis of metabolites in the bioreactor supernatant showed a gradual glucose consumption over time during exponential cell growth till day 10 when growth plateaued (Fig. 5c). Glutamine, being an essential energy source and nitrogen donor for cells *in vitro*, was excluded from our media to put selection pressure on cells. This helps to ensure that only cells with high expression of GLUL and subsequent production of EPO would be able to survive. Glutamine concentration, although starting at 0 mM, was observed to increase over time from day 4 and reached a peak of 3.3 mM at day 9 (Fig. 5d). Since culture viability was above 88% throughout the 10-day culture, we postulate that the increase in glutamine levels was due to the increase in cell density and as such GLUL concentrations in the bioreactor over time. This may have resulted in increased conversion of glutamate into glutamine by GLUL above that needed by the cells to result in increased glutamine concentration. Evidence that this glutamate to glutamine conversion is essential for *GLUL*^−/−^ cells to grow was observed at day 7 of culture where exponential growth of the cells almost depleted the supply of glutamate in the media (Fig. 5e). Since glutamate is one of the limiting factors in the growth of *GLUL*^−/−^ cells, this deficiency was rectified via the addition of feed media to maintain cell growth and viability. Accumulation of growth-inhibiting metabolites, lactate and ammonia, were minimal in our bioreactor runs, reaching maximum values of 1.5 g/L and 0.8 mM respectively (Fig. 5f,g). In contrast, consumption of lactate and ammonia was observed on day 4 and day 7 respectively. The consumption of ammonia coincided with the decrease in glutamate and increase in glutamine, suggesting that glutamate is being converted to glutamine due to the GLUL expression. This is consistent with previous report^33^. Osmolality was also maintained at physiological levels (between 277 and 302 mOsm/kg) during the 10 days duration of the run (Fig. 5h). These data suggest that growth and production of EPO during the bioreactor run was not hindered by the analyzed metabolites.

### Glycosylation analysis of HEK293-derived EPO

**Figure 6:**
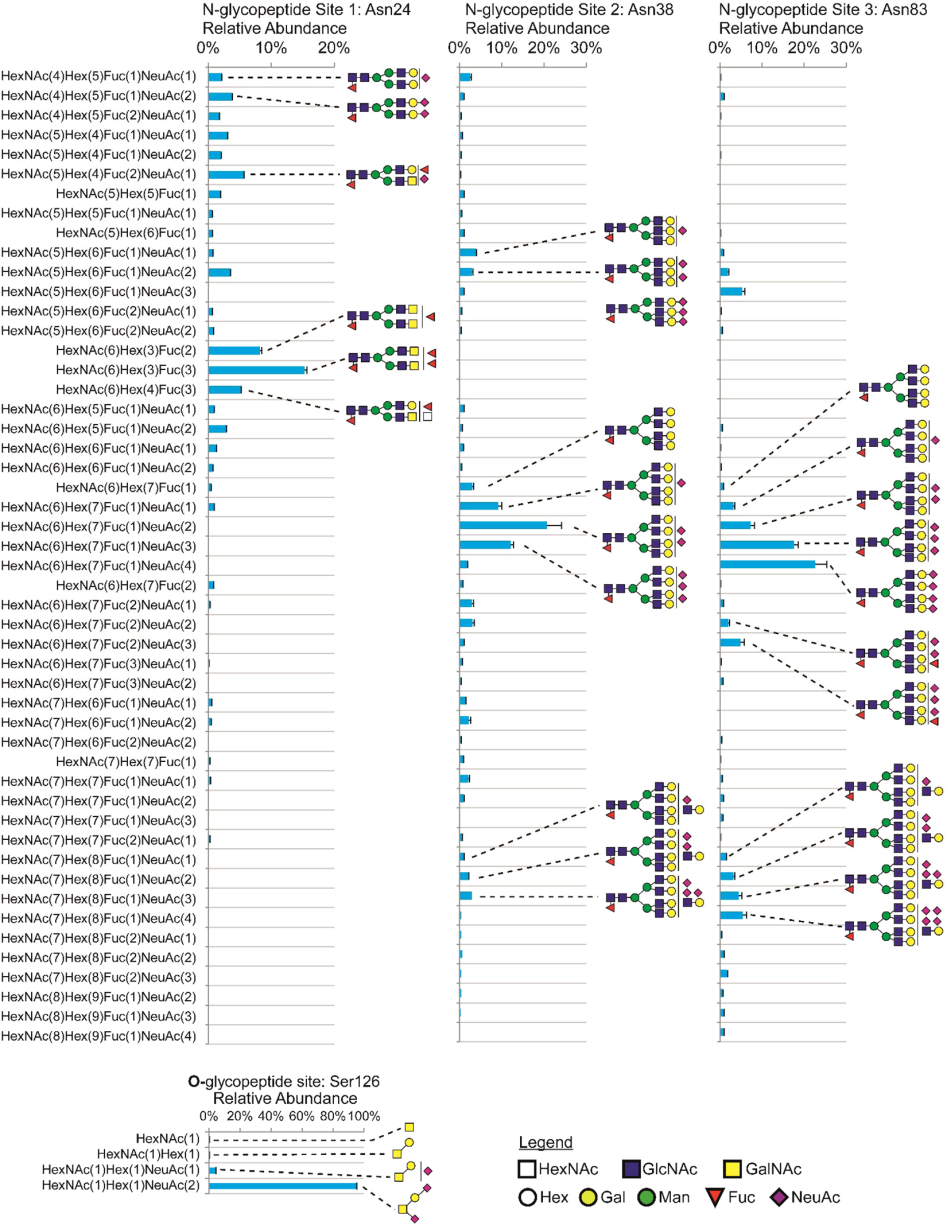
HEK293-derived EPO site-specific N-glycosylation. Glycopeptides from all three N-glycan sites were acquired with LCMS/MS Orbitrap HCD (normalized collisional energy 30%) and identified using the Byonic software. Label-free quantitation was done using OpenMS/KNIME.

To understand the glycosylation micro- and macroheterogeneity of the EPO produced from the HEK-EPO cell pool, site-specific glycopeptide analysis of the EPO harvested from Day 10 of the bioreactor culture was carried out (Fig. 6). Depicted are relative abundances determined by integrating the extracted ion chromatograms for each glycopeptide measured on the mass spectrometer. The HEK-derived EPO was heavily sialylated on both N-glycosylation and O-glycosylation, with on average 6.55 NeuAc per mole of EPO (Table 2). This degree of sialylation was similar to that of EPO produced in CHO^34^, with an average 6.6 sialic acids per mole of EPO. Notably, no alpha-Gal or Neu5Gc was detected in our HEK-derived EPO, whereas Neu5Gc was found in 4.7% of glycopeptide spectra in CHO-derived EPO^34^.

**Table 2:** Summary of glycosylation of HEK293-derived EPO.

Glycosylation site (residue)	Core fucosylation (mol core Fuc/mol EPO)^§^	Sialylation (mol NeuAc/mol EPO)^§^	Antennae (% at each site) [bi/tri/tetra]^‡^	Site Occupancy (%)*
Asn24 N-glycan	0.9832	0.58	86.85/8.11/5.04	100.00
Asn38 N-glycan	0.9965	1.70	68.14/10.11/21.75	100.00
Asn83 N-glycan	0.9954	2.82	64.06/9.09/26.85	99.96
Total N-glycosylation	2.9751	5.10	73.02/9.11/17.88	99.99
Ser126 O-glycan	Not applicable	1.45	Not applicable	74.45
Overall glycosylation	Not applicable	6.55	Not applicable	93.60

^‡^Abundances of bi-/tri-/tetra-antennary structures were normalized for each N-glycan site. Antenna % for Total N-glycosylation was the average of the three sites’ normalized abundances.

^§^Moles of glycan per EPO were calculated by summing the number of NeuAc in each composition and multiplying by the relative abundance of the glycopeptide (normalized over each N- and O-glycosylation sites). The Total-N-glycosylation was calculated as the sum of the site specific mole ratios.

^*^Site occupancy was defined as 100% minus the relative abundance of the unglycosylated peptide normalized for each glycan site.

The degree of sialylation was different across the sites, with Site 3> Site 2> Site 1. The higher sialylation at Site 2 and Site 3 corresponded with these two sites having more tetra-antennary N-glycans than Site 1 and hence more substrates for sialylation by sialyltransferases. The single O-glycan site was dominated by the doubly (NeuAc2GalGalNAc) sialylated Core 1 O-glycan (Fig. 6), similar to CHO-derived EPO^34^. On average, EPO N-glycans were almost fully core fucosylated, with 2.98 moles out of a possible 3.00 moles of core fucose per moles EPO. The EPO glycosylation site occupancies were evaluated by determining the ratio of the abundance of the non-glycosylated peptides and the sum of their differently glycosylated counterparts. From this analysis, it was observed that EPO was completely N-glycosylated at Site 1 and Site 2 and at 99.96% occupancy at Site 3 (Table 2).

## Discussion

The use of human cell lines for production of recombinant proteins is increasing with many currently in development and some already approved. In this study, we used a *GLUL*^−/−^ HEK293 cell line with a human *GLUL* selection marker and MSX selection to successfully generate an EPO cell line producing high titer of EPO without non-human glycan epitopes. The use of the GLUL-MSX system abrogates issues with glutamine supplementation since glutamine is unstable and breaks down into ammonium and pyroglutamate, which at elevated concentrations results in inhibited cell growth, reduced terminal sialylation and increased glycoform heterogeneity^35,36^. Higher initial concentrations of glutamine has also been shown to increase ammonia concentrations in both CHO-DG44 and HEK293E cell cultures^37^. Our choice for disrupting the *GLUL* gene is to allow for regulated synthesis of glutamine and thereby ammonia, wherein eventual GLUL-transfected cells can be cultured in a glutamine-deficient media. Subsequently, *GLUL* expression can be selected with MSX to enhance the productivity of HEK293 cells to yield high titers of EPO. Compared to DHFR-MTX amplification, the GLUL-MSX selection system requires only a single round of selection to obtain high-expressing producer cells^38^. In this study, selection with 100 nM was sufficient to achieve high titer of 92700 U/mL of EPO as analyzed by ELISA or 696 mg/L by densitometry. Further increase in yield can potentially be achieved through the addition of sodium butyrate^39^ or through a temperature shift from 37 °C to 33 °C at the stationary phase of culture (unpublished data).

Sialylation of N-glycosylation greatly reduces clearance from the blood^40^. The high level of sialylation of EPO produced in HEK293 indicates that its serum half-life will be comparable to the CHO version. Glycosylation analysis also showed that Site 1 had lower degree of branching than Sites 2 and 3, which is consistent with previous report on EPO produced in CHO cells^34^. The similar pattern between CHO and HEK293 suggested that in both cell lines the MGAT4 and MGAT5 glycosyltransferases that add additional antennae may be sterically blocked from accessing Site 1 but not Sites 2 and 3. The similar patterns and levels of sialylation and N-glycan branching between the HEK293 derived EPO in this study and EPO from CHO suggest that EPO is subjected to similar kinds of processing in both cell lines. Importantly, these similarities also indicate that the GLUL-mediated gene selection strategy did not interfere with the glycosylation machinery but maintained the glycosylation occupancy of EPO.

It should be noted that the LacdiNAc glycan epitopes on EPO are expected for recombinant glycoproteins produced in HEK293 because this cell line expresses an active β1-4 N-acetylgalactosaminyltransferase^41^. LacdiNAc is present on other glycoproteins expressed in HEK293 such as glycodelin and is not immunogenic as it is present in humans^42^. HEK293 is therefore able to glycosylate EPO with human glycans. Although the therapeutic implications of the higher N-glycan heterogeneity and sialylated and fucosylated LacdiNAc in EPO are not clear, these epitopes are emerging as ligands for the galectin-3, a carbohydrate binding protein secreted by immune cells and kidney cells and that can form galectin-glycoprotein lattices on the cell surface^43–45^. Potential binding to galectin-3 suggests that there may be a positive effect on the pharmacokinetics of EPO, such as increased half-life due to being in complex with galectin-3.

Use of human cell lines for recombinant protein production is steadily expanding with properties such as adaptability in serum-free cultures, scalability and high productivity being comparable to current non-human producer cell lines such as CHO and NS0. More importantly, human cellular expression systems do not produce immunogenic Neu5Gc and α-Gal epitopes which are known to impede safety and therapeutic efficacy. Human cells also have an added advantage in its capacity to produce human PTMs required for activity by complex recombinant proteins. With continued efforts in further developing human cell lines for biomanufacture, preference for a suitable host platform in the near future may transition from non-human to human cellular expression systems.

## Methods

### Cell lines and expression vectors

HEK293 cells (ATCC^®^ CRL1573^™^) were cultured in DMEM/F-12 (Thermo Fisher Scientific, Gibco^™^, Waltman, MA, USA) supplemented with 10% dialyzed FBS (Thermo Fisher Scientific, Gibco^™^) in adherent format. Knockout of glutamine synthetase (*GLUL*) gene by CRISPR-Cas9 was then carried out to generate four parental knockouts, GLUL^−/−^ #7, #20, #24 and #29. Human erythropoietin (*EPO*) and human glutamine synthatase (*GLUL*) precursor genes were codon-optimized (Genscript, Piscataway, NJ, USA) and cloned into a bicistronic IRES expression vector.

### Generation of *GLUL*-KO HEK293 cell lines via CRISPR/Cas9 system

HEK293 cells were seeded at 0.35 × 10^6^ in a 6-well plate one day prior to transfection, to ensure ~70–80% confluency the next day. Cas9-expressing plasmid (6 µg) was co-transfected with 3 µg sgRNA1-expressing and 3 µg sgRNA2-expressing plasmids using Turbofect transfection reagent (Thermo Fisher) according to the manufacturer’s instructions. Successfully transfected cells were sorted by flow cytometry and plated sparsely on a 10 cm culture plate. Subsequently, individual clones were picked and seeded into 96-well plates (1 clone/well), and left to grow till confluency before expanding into 48-well plates. When grown to confluency, 30% of the cells were used for gDNA extraction, while 70% were re-seeded.

### Sequencing of targeted *GLUL* genomic locus

Genomic DNA was isolated from potential *GLUL*-KO cell lines using DNeasy Blood & Tissue Kit (Qiagen, Hilden, Germany). PCR using KOD Hot-start (Merck-Novagen, Darmstadt, Germany) was then performed to amplify *GLUL* with primers F: 5′-CTCCAGAACACCTTCCACCA-3′ and R: 5′-ACATTGCTGTCTCACCTTCC-3′. Cycling conditions were programmed as follows: 95 °C for 2 min, followed by 35 cycles of 95 °C for 20 s, 55 °C for 10 s, and 70 °C for 15 s. Further extension was carried out with Taq polymerase (Thermo Fisher Scientific) to add 3′ adenine overhangs for cloning into a T-vector containing complementary thymidine residues. PCR products were run on a gel where single bands obtained were extracted using Gel Extraction Kit (Machery-Nagel, Düren, Germany). Cleaned PCR products were then transformed in One Shot® TOP10 Chemically Competent *E*. *coli* (Thermo Fisher Scientific) using a TOPO TA cloning kit (Thermo Fisher Scientific, Invitrogen) before plating on LB agar plates (2.5% LB broth miller, Merck-Novagen and 1.5% BD Bacto™ Agar, Becton, Dickinson and Company, NJ, USA). Colonies were grown in 3 mL LB broth culture (2.5% LB broth miller, Merck-Novagen) before plasmid extraction (QIAprep Spin Miniprep Kit, Qiagen). Purified plasmids were subsequently sequenced via the BigDye® Terminator v3.1 cycle sequencing kit carried out by 1^st^ Base DNA Sequencing service (Singapore).

### Transcriptome analysis by RNA-seq

High throughput sequencing libraries were generated using the NEB Next Ultra II Directional RNA Library Prep Kit for Illumina together with the NEBNext Poly(A) mRNA Magnetic Isolation Module (New England Biolabs), according to manufacturer’s instructions. Sequenced reads were mapped to human genome assembly hg19 using STAR with maximum number of mismatches allowed per pair (outFilterMismatchNmax) assigned to 6 and maximum number of mismatches per pair relative to read length (outFilterMismatchNoverLmx) assigned to 0.05. DESeq2 package was used to calculate the gene expression counts and perform the differential gene expression calculations. Correction of batch effects was performed using ComBat.

### Development of EPO-producing HEK293 cells

*GLUL*^−/−^ cells were adapted to suspension culture in a DMEM/F12-based protein free-chemically defined media (PF-CDM) supplemented with 1 × non-essential amino acids (NEAA) (Thermo Fisher Scientific, Gibco^™^). Stable pools were generated via electroporation using the SG Cell Line kit in a 4D-Nucleofector system (Lonza, Basel, Switzerland). AhdI-linearized EPO expression vector (4 µg) containing *EPO* and *GLUL* genes (Uniprot IDs P01588 and P15104 respectively) was transfected into 3 × 10^6^ suspension *GLUL*^−/−^ cells. Selection involved distribution of 10,000 cells per well into a 96 well plate containing glutamine-deficient DMEM/F-12 medium supplemented with 10% dialyzed serum, with further selection by supplementing with 100 nM of methionine sulfoximine (MSX) (Merck Millipore, Burlington, MA, USA). Supernatant from growing wells were tested for EPO titer using EPO Human ELISA Kit (Thermo Fisher Scientific, Invitrogen). The highest producers were scaled up sequentially into a 125 mL shake flask suspension culture in PF-CDM. Five cell pools from *GLUL*^−/−^ #7, three cell pools from *GLUL*^−/−^ #24 and one cell pool from *GLUL*^−/−^ #20 survived the adaptation to serum free, glutamine-deficient, PF-CDM media containing 100 nM MSX. Surviving cell pools were then subjected to growth, productivity and stability testing analysis as described below.

### Batch cell cultures

Routine suspension culture involved passaging every 3–4 days in 125 mL flasks (Corning, Corning, NY, USA) and growth in a shaking incubator (Kuhner, Birsfelden, Switzerland) at 37 °C, 8% CO2 and 110 RPM.

Growth and productivity analysis was carried out in a shake flask batch culture of 40 mL volume at 0.5 × 10^6^ cells/mL seeding density in PF-CDM containing MSX. Sampling was carried out daily throughout the culture for analysis of viable cell density, viability, EPO titer and biochemical profile.

Stability testing was carried out by culturing the cells for 12 weeks in shake flasks at 0.5 × 10^6^ cells/mL seeding density in 20 mL of PF-CDM without MSX. During the 12 weeks, flasks were passaged twice a week and day 4 supernatants were collected for titer analysis by ELISA.

### Fed-batch culture

EPO-producing HEK293 cells were scaled up in PF-CDM supplemented with animal-free Proyield Soy SE50MK-NK hydrolysate (FrieslandCampina, Amersfoort, Netherlands) and inoculated into duplicate 2 L glass bioreactors (Sartorius, Göttingen, Germany) at a VCD of 0.5 × 10^6^ cells/mL. Culture temperature, pH, dissolved oxygen and stir-rate were maintained at 36.5 °C, 7.0, 40% and 150 rpm respectively. Culture glucose and glutamate were topped up daily to 4 g/L and 3 mM respectively by feeding with a concentrated protein-free feed containing glucose and a 500 mM glutamate solution.

### Analysis of cell culture samples

To assess the health and growth of cells in culture, 0.5 mL of suspension cell culture was sampled on a Vi-CELL™ Cell Viability Analyzer (Beckman Coulter, Brea, CA, USA) to automatically analyse 50 images per sample using trypan blue exclusion to provide data on live cell counts and percentage viability. In addition, other key characteristics tracked during growth profiling of cell pools included pH, glutamine, glutamate, glucose, lactate, ammonia, sodium, potassium and osmolality. These concentrations were analysed using a BioProfile® 400 Analyzer (NOVA biomedical, Waltham, MA, USA) from 1 mL of cell-free supernatant, clarified by centrifugation at 8,000 rpm for 10 mins.

### Titer analysis

Protein productivity was tracked by regular titer analysis of cell culture supernatant using a short incubation EPO Human ELISA Kit (Thermo Fisher Scientific). Samples were diluted to a detectable range of 1.6–100 mIU/mL, before being loaded in duplicates and tested for EPO titer according to manufacturer’s instructions.

For densitometry, a mg/L titer was first estimated from the U/mL titer obtained from ELISA using an estimated conversion factor of 150 U/µg based on the 3^rd^ WHO International Standard for Erythropoietin (National Institute for Biological Standards and Control, NIBSC, Herts, UK). With this mg/L concentration, an estimated amount of EPO from each sample was run on an SDS-PAGE gel together with a commercial EPO standard from Abcam (ab215737) at different EPO loadings. The gel was then stained by Coomassie Brilliant Blue R-250 (Thermo Fisher Scientific). Intensities of each band in our gel were evaluated using ImageJ software by densitometry and compared with the standard curve generated with the Abcam EPO standard to obtain the percentage Calculated / Loaded EPO amount (Supplementary Fig. 1). These percentages were then multiplied with the mg/L titers that were estimated from ELISA U/mL titers based on 150 U/µg, to obtain the densitometry EPO titers in mg/L.

### Expression analysis

#### Droplet digital PCR (ddPCR) analysis

Genomic DNA (gDNA) or RNA was extracted from HEK293 wildtype, *GLUL*^−/−^ clone #24 and EPO-producing cell pool #8 cultured with 100 nM MSX. Droplet digital PCR was performed using a QX100 Droplet Digital PCR system (Bio-Rad, Hercules, CA, USA). For gene copy analysis, a total volume of 20 μl reaction mix containing 2× QX200 ddPCR Evagreen supermix (Bio-Rad), 150 nM of primers (sequences shown in Supplementary Table 1), and 50 ng of gDNA in nuclease-free water was prepared. To perform mRNA ddPCR analysis, 500 ng of RNA was first converted into cDNA using iScript Reverse Transcription Supermix (Bio-Rad). Complementary DNA (2 μl) was mixed into the ddPCR reaction as per gene copy analysis.

Samples were then transferred to the DG8 cartridge, together with 70 μl of oil into its lower wells, before being loaded into the QX200 droplet generator (Bio-Rad). The droplet mixture (40 μl) from each sample was transferred to a 96-well PCR plate, sealed with the Bio-Rad PX1 PCR plate sealer and inserted into the C1000 thermal cycler (Bio-Rad). Cycling conditions using EvaGreen assay were programmed as follows: 95 °C for 5 min, 95 °C for 40 cycles at 30 s per cycle 58 °C for 1 min, followed by 4 °C for 10 min and 90 °C for 5 min. A ramp rate of 2 °C/s was used for the entire cycling process. Following, the plate was assessed in the QX200 droplet reader for completed PCR reactions in individual droplets and the data was analyzed using the QuantaSoft software (Bio-Rad). Thresholds for determining positive droplets were established manually based on wells containing water alone which served as negative controls. The number of copies of the gene of interest per ng of input gDNA or RNA was then calculated as below.

$${\rm{Copies}}\,{\rm{per}}\,{\rm{ng}}\,{\rm{gDNA}}\,{\rm{or}}\,{\rm{RNA}}={\rm{Copies}}\,{\rm{per}}\,20\mu l/{\rm{Input}}\,{\rm{gDNA}}\,{\rm{or}}\,{\rm{RNA}}\,{\rm{amount}}$$

To determine genomic copy count, *PDHA2* was used as a reference locus, where number of copies in HEK293 wildtype genome was determined to be 2. Other genes characterized relative to *PDHA2* include *PDHA1* and *SERPINA1* which both contain 3 copies each in the HEK293 wildtype genome, synonymous to a previous study^46^. The following formula was then used to determine genomic copy count of target genes:

$${\rm{Genomic}}\,{\rm{copy}}\,{\rm{per}}\,{\rm{cell}}=({\rm{Copies}}\,{\rm{per}}\,{\rm{ng}}\,{\rm{gDNA}}\,{\rm{of}}\,{\rm{target}}/{\rm{Copies}}\,{\rm{per}}\,{\rm{ng}}\,{\rm{gDNA}}\,{\rm{of}}\,PDHA2\,{\rm{reference}})\times 2$$

#### Western blotting

To detect GLUL by immunoblotting, cell pellets were first homogenized in phosphatase inhibitor lysis buffer. Lysed cell pellets were then diluted in LDS sample buffer and heated in reducing agent prior to loading onto the gel for Western blotting as previously described^29^. To detect EPO, culture supernatant were similarly diluted and reduced prior to loading and immunoblotting. Blots were incubated with antibodies: anti-EPO (1:1000; MAB2871, R&D systems, MN, USA), anti-GLUL (1:5000; MAB302, Merck Millipore) and anti-actin (Fig. 1b: sc-47778, Santa Cruz, RX, USA; Figs 1f and 4c: 1:6000; A2066, Sigma-Aldrich, MO, USA). Figure 1b was imaged using ChemiDoc (Bio-Rad), while Figs 1f and 4c were imaged on a chemiluminescent CCD camera, ImageQuant LAS 500 (GE, MA, USA). Full length gel images are shown in Supplementary Table and Figures File.

### Glycosylation analysis

HEK-derived EPO was harvested from a bioreactor fed-batch culture (Fig. 5, Reactor 1) on day 10 at a viable cell density of 10.8 million cells/ml and culture viability of 89.9%. A two-step purification protocol was performed consisting of ion exchange chromatography followed by size exclusion chromatography on an ÄKTA Purifier 10 FPLC system (GE Healthcare) connected to Frac-950 (GE Healthcare). Culture supernatant was adjusted to pH of 8.0 and conductivity of 4 mS/cm before loading onto a HiTrap Capto Q (GE Healthcare) column pre-equilibrated with 50 mM Tris, pH 8.0. Column was subsequently washed with the same equilibration buffer before elution of EPO at 150 mM NaCl in 50 mM Tris, pH 8.0 and flow rate of 1 mL/min. Peak fractions were collected and verified to contain EPO by SDS-PAGE prior to concentration with Amicon Ultra-15 10 K (Merck-Millipore). The concentrated fraction was then injected into a HiLoad 16/600 Superdex 75 pg (GE Healthcare) equilibrated with 1 x PBS buffer. Utilizing a 1 mL/min flow rate of PBS buffer, peak fractions were collected and combined before determining purified titer using an EPO ELISA kit (ThermoFisher Scientific). The purified EPO were denatured reduced, alkylated with iodoacetic acid and digested with trypsin (1:50 weight ratio, sequencing grade, Promega, WI, USA) and GluC (1:50 weight ratio, sequencing grade, Promega), using a filter aided sample preparation method described previously^47^.

#### nanoLC-MS analysis of samples

Approximately 2.5 µg of glycopeptides were injected (Dionex Ultimate WPS-3000 RSLCnano) and desalted on a C18 PepMap100 trap column (5 µm particle size, 10 nm pore size, 300 µm I.D., 5 mm length, Thermo Fisher Scientific) and separated on a nanoflow EASY-Spray PepMap RSLC C18 column (2 µm particle size, 10 nm pore size, 50 µm internal diameter, 150 mm length, Thermo Fisher Scientific) at 40 °C using a 104 min linear gradient from 2% to 50% acetonitrile in LCMS-grade water (Merck) with 0.1% formic acid (>98% purity, Merck) at 300 nL/min. The eluting peptides were analysed with an Orbitrap Fusion Tribrid (Thermo Fisher Scientific) with the following parameters: spray voltage at +2000 V, ion transfer tube at 300 °C, with no sweep gas. Full profile scans used the Orbitrap at 120,000 resolution, *m/z* 300–3000 range with a maximum injection time of 70 ms, using quadrupole isolation. MS/MS was triggered for peptides of charge state 2–6, intensity more than 5.0e4 and apex detected as 35% of the full width at half maximum (FWHM) of a gaussian peak of 12 seconds FWHM. An exclusion list was constructed by combining all the peaks from the negative control gel pieces, a blank run and BSA. Dynamic exclusion of 30 seconds was applied. MS/MS fragmentation done using higher collisional dissociation (HCD) with normalized stepped collisional energy of 30 ± 5% and fragments were detected with the Orbitrap operating at 30,000 resolution and a maximum injection time of 100 ms.

#### Glycoproteomic data analysis

LCMS data files were analyzed by commercial software Byonic (ProteinMetrics ver. w2.14–27) and aided by the GlycopeptideGraphMS software^48^. Data files were imported into Byonic and the following search settings were used: fully specific cleavage C-terminal of arginine, lysine, aspartic acid and glutamic acid, three missed cleavages, precursor mass tolerance 10 ppm, fragment mass tolerance 10 ppm, up to two common modifications were allowed of variable methionine oxidation, fixed cysteine carboxymethylation, up to one rare modifications was allowed from either one N-glycan from a library developed for HEK293, or one O-glycan from the 6 most common structures, and the protein list was the SWISSPROT database. In this way, glycopeptides were identified by searching their MS/MS fragmentation for oxonium ions, peptide sequence ions (b- and y-ions) and glycan neutral losses (B- and Y-ions) as well as the complete intact mass of the glycopeptide.

The abundances of identified glycopeptides were computed using an OpenMS pipeline, which involved integrating the area under the extracted ion chromatogram for all detected charge states, grouping them into LCMS features, and deconvoluting^49^. Redundant glycopeptides (i.e. spanning the same glycan site but with missed cleavages) were grouped together for site-specific analyses. LacdiNAc was deemed to be present if either the composition allowed for pairs of unoccupied HexNAc after placing all non-core Hex in LacNAc antennae or if the *m/z* 407.1658 oxonium ion [HexNAc2 + H]^+^ was present. The glycan features of EPO were quantified as follows: the first fucose was always assigned as core fucosylation and the relative abundances (normalized to overall N-glycosylation) of glycopeptides with core fucose was summed and divided by 100 to give the mol core fucose / mol EPO; sialylation and total fucosylation was derived by multiplying the relative abundance (normalized to overall N-glycosylation) by the number of NeuAc, then summing the results; branching was calculated per site by separately summing the abundances of mono-, bi-, tri- and tetra-antennary structures; site occupancy was calculated as 100% minus the relative abundance (normalised to each site) of the unglycosylated peptide.

### Calculations

Specific growth rate (µ) was determined by plotting ln(VCD) vs *t* according to Eq. 1, where VCD is the viable cell density, VCD_0_ is the initial viable cell density and t is the culture time.

1 $$\begin{array}{c}{\rm{VCD}}={{\rm{VCD}}}_{0}{{\rm{e}}}^{\mu {\rm{t}}}\\ \mathrm{ln}({\rm{VCD}}/{{\rm{VCD}}}_{0})=\mu {\rm{t}}\end{array}$$

The cumulative integrated viable cell density (IVCD) was calculated by trapezium rule according to Eq. 2.

2 $${{\rm{IVCD}}}_{{\rm{t}}}={{\rm{IVCD}}}_{{\rm{t}}-1}+0.5\times ({{\rm{VCD}}}_{{\rm{t}}}+{{\rm{VCD}}}_{{\rm{t}}-1})\times {\rm{\Delta }}{\rm{t}}$$

Specific EPO productivity (q_p_) between culture times t_1_ and t_2_ was determined by plotting IgG titer (P) vs IVCD according to Eq. 3.

3 $$\begin{array}{c}{{\rm{q}}}_{{\rm{p}}}=({{\rm{P}}}_{{\rm{t}}2}\mbox{--}{{\rm{P}}}_{{\rm{t}}1})/({{\rm{IVCD}}}_{{\rm{t}}2}-{{\rm{IVCD}}}_{{\rm{t}}1})\\ {{\rm{P}}}_{{\rm{t2}}}={{\rm{q}}}_{{\rm{p}}}\times ({{\rm{IVCD}}}_{{\rm{t}}2}-{{\rm{IVCD}}}_{{\rm{t}}1})+{{\rm{P}}}_{{\rm{t}}1}\end{array}$$

## Supplementary information

Supplementary Table and Figures

Supplementary File 1 - WT vs KO

Supplementary File 2 - Adherent vs Suspension

## Data Availability

The RNA-seq data generated in the current study will be deposited in the NCBI GEO database and made publicly available upon acceptance of the manuscript.
