# Supplementary material for: A human expression system based on HEK293 for the stable production of recombinant erythropoietin
Source: Sci Rep. 2019 Nov 14;9:16768. doi: 10.1038/s41598-019-53391-z (PMC6856173; doi:10.1038/s41598-019-53391-z)

A human expression system based on HEK293 for the stable production of recombinant erythropoietin

Christine Chin[1,+], Justin Bryan Goh[1,+], Harini Srinivasan[2,3], Kaiwen Ivy Liu[3], Ali Gowher[3], Raghuvaran Shanmugam[3], Hsueh Lee Lim[1], Matthew Choo[1], Wen Qin Tang[1], Andy Hee-Meng Tan[1], Terry Nguyen-Khuong[1], Meng How Tan[2,3,]*, Say Kong Ng[1,]*

[1]Bioprocessing Technology Institute, Agency for Science, Technology and Research (A*STAR), Singapore; [2]School of Chemical and Biomedical Engineering, Nanyang Technological University, Singapore; [3]Genome Institute of Singapore, Agency for Science, Technology and Research (A*STAR), Singapore.
[+] These authors contributed equally to the work.

* Corresponding authors:
MHT: School of Chemical and Biomedical Engineering, Nanyang Technological University, 62 Nanyang Drive, N1.2-B2-33, Singapore 637459, Singapore. Telephone: 65-65138063, Fax: 65-67947553, Email: mh.tan@ntu.edu.sg

SKN: Bioprocessing Technology Institute, Agency for Science, Technology and Research (A*STAR), 20 Biopolis Way, #06-01, Centros, Singapore 138668, Singapore. Telephone: 65-64070919, Fax: 65-64789561, Email: ng_say_kong@bti.a-star.edu.sg

Supplementary Table and Figures

Supplementary Table 1. Primers used for the quantification of gDNA or mRNA copy numbers of WT and vector *GLUL* and *EPO* genes.

**PCR target**	**Primers**	**Sequence**	**Amplicon size (bp)**	**Targeting template**
WT *GLUL*	Forward	5’-TGTATATCTGGATCGATGG-3’	96	gDNA
	Reverse	5’-ACATTGCTGTCTCACCTTC-3’		
WT *GLUL*	Forward	5’-AAAGAGGGCAACCCTAAC-3’	170	cDNA
	Reverse	5’-GATCGATGGTACTGGAGAA-3’		
Vector *GLUL*	Forward	5’-TGCTGGAGTGAAGATTGCAG-3’	179	gDNA and cDNA
	Reverse	5’-CTGGAATAGGCTTGGGATCA-3’		
WT *EPO*	Forward	5’-CCTGTTTTCGCACCTACCAT-3’	143	gDNA and cDNA
	Reverse	5’-GTCTTCATGGTTCCCACCAC-3’		
Vector *EPO*	Forward	5’-ACAGCAGGCAGTGGAAGTCT-3’	193	gDNA and cDNA
	Reverse	5’-TGGTGAGATGGCCTCTTTCT-3’		

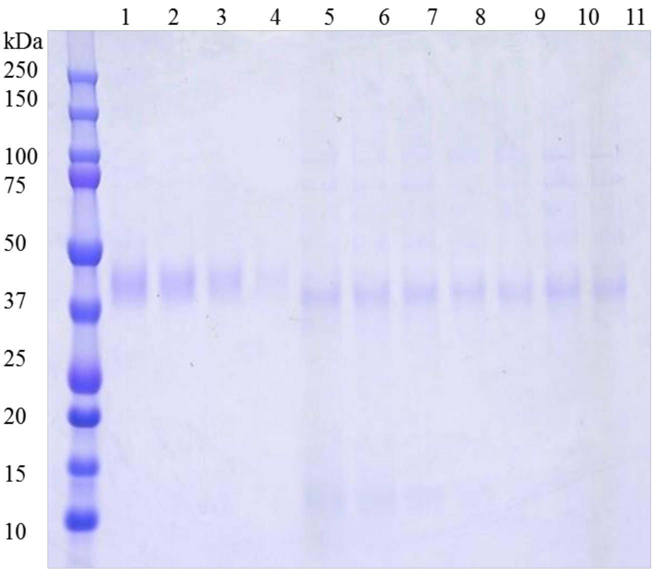

Lane number	Sample	Loaded EPO amount (µg)	Area Value (from image J)	Percent Value (from image J)	Calculated EPO amount obtained from standard curve (µg)	Calculated / Loaded EPO amount (%)
1	Abcam 215737 Std	1	81897	15.28	0.99	99%
2	Abcam 215737 Std	0.75	61524	11.48	0.77	103%
3	Abcam 215737 Std	0.5	37316	6.96	0.47	94%
4	Abcam 215737 Std	0.25	22770	4.25	0.26	106%
5	Reactor Day 4	0.68*	57883	10.80	0.73	108%
6	Reactor Day 5	0.68*	41525	7.75	0.53	78%
7	Reactor Day 6	0.68*	40640	7.58	0.52	76%
8	Reactor Day 7	0.68*	36054	6.73	0.45	67%
9	Reactor Day 8	0.68*	41156	7.68	0.52	77%
10	Reactor Day 9	0.68*	54681	10.20	0.69	102%
11	Reactor Day 10	0.68*	60688	11.32	0.76	113%

*estimated conversion based on 150 U/µg, as per the 3rd International Standard by the National Institute for Biological Standards and Control (NIBSC code: 11/170)

Supplementary Fig. 1. Comparison of EPO titer with commercial EPO standard. Commercial EPO standard (Abcam 215737) was loaded onto a SDS-PAGE gel in amounts ranging from 1 µg to 0.25 µg to determine band intensity in comparison to mass. Reactor samples from day 4 to day 10 had titers that were previously evaluated by ELISA (Figure 5b). From these titers, 0.68 µg of EPO for each reactor sample was estimated using a 150 U/µg conversion factor and loaded on the same SDS-PAGE gel. After staining with Coomassie Brilliant Blue R-250, band intensities between commercial EPO standard and reactor sample EPO were compared using Image J software. Area Value was established as the area under peak of each band whereas Percent Value was the percentage of one peak compared to all other peaks. A standard curve was established using the Percent Value of the commercial EPO standard and the amount loaded. This was used to calculate the amount of EPO in the reactor samples loaded on the gel. The calculated EPO amount was then compared with the estimated EPO loading to derive a percentage that is used as a correction factor to determine the titer of EPO in the reactor samples in mg/L.

Supplementary Information: Full length gel images

Samples are separated on an SDS-PAGE gel for each figure as described in Methods. The gels were then cut and immunoblotted with the respective antibodies separately. (a) Gel images for Fig. 1b. (b) Gel images for Fig. 1f. (c) Gel images for Fig. 4c.

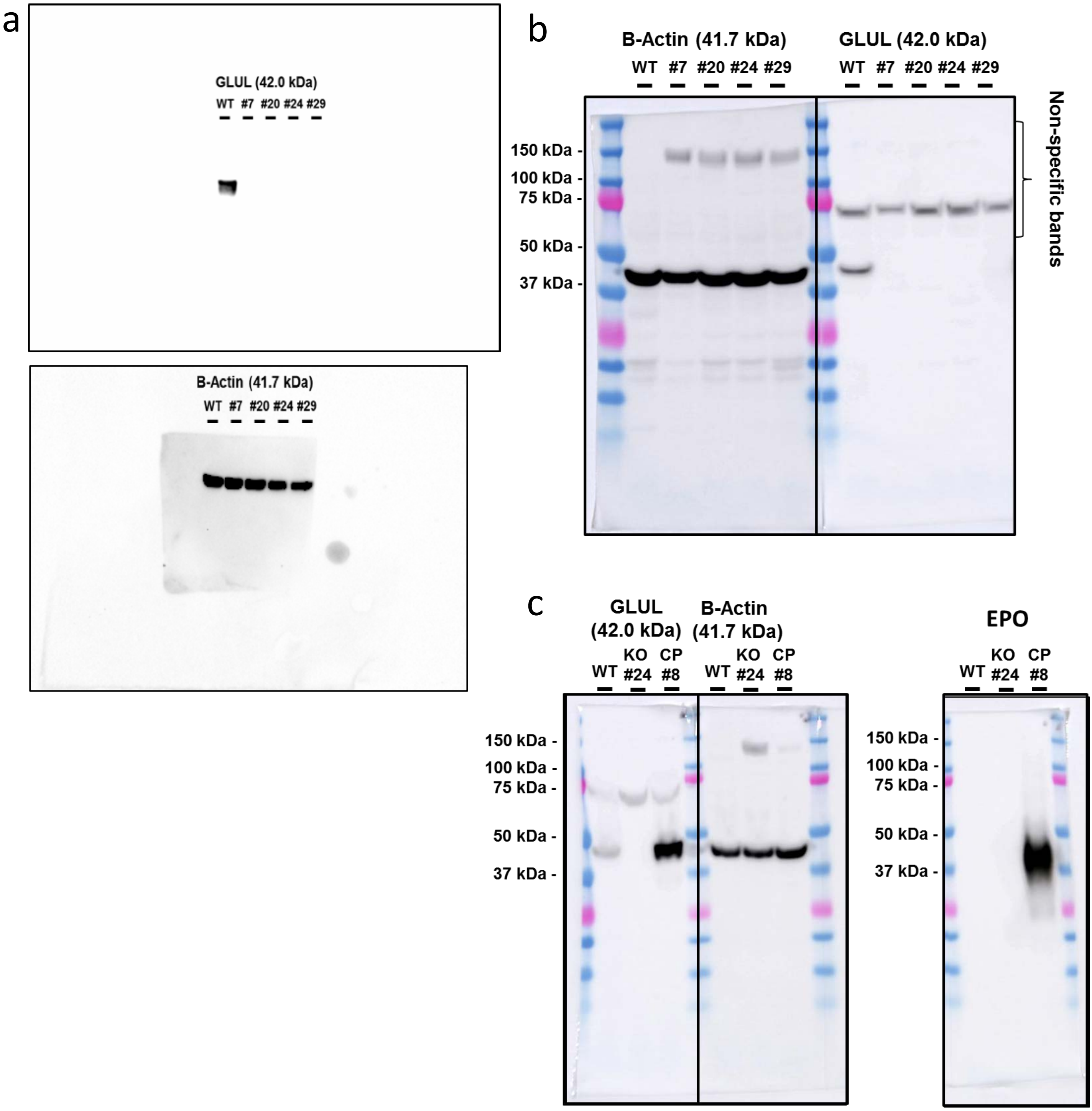

Supplement: Supplementary file 1 — Supplementary Table and Figures [file 41598_2019_53391_MOESM1_ESM.pdf]
